# Editorial: Human factors and cognitive ergonomics in advanced industrial human-robot interaction

**DOI:** 10.3389/frobt.2025.1564948

**Published:** 2025-02-28

**Authors:** Erik Billing, Federico Fraboni, Luca Gualtieri, Patricia Helen Rosen, Peter Thorvald

**Affiliations:** ^1^ Interaction Lab, School of Informatics, University of Skövde, Skövde, Sweden; ^2^ Department of Psychology, Alma Mater Studiorum, University of Bologna, Bologna, Italy; ^3^ Faculty of Engineering, Free University of Bozen-Bolzano, Bolzano, Italy; ^4^ Unit Human Factors and Ergonomics, Federal Institute for Occupational Safety and Health, Dortmund, Germany; ^5^ User Centred Product Design, School of Engineering Science, University of Skövde, Skövde, Sweden

**Keywords:** cognitive ergonomics, human factors, human-robot collaboration, human-robot interaction, industry 5.0

## 1 Introduction

Collaborative robotics is a very promising technology for many industrial processes, including e.g., manufacturing, logistics, or construction. This new technology are also changing the environment for workers in industry. Research on human-robot interaction (HRI) will be crucial for enhancing the operator’s work conditions and wellbeing, as well as production performance. In that regard, human factors, with a special emphasis on cognitive ergonomics are fundamental to implementing safe, fluent, and efficient collaborative applications.

This Research Topic gathers a range of contributions on the study of Human Factors and Cognitive ergonomics in user-centered and collaborative applications in industrial settings. Here, we summarize these studies from the perspective of three pivotal areas impacted by collaborative robotics: workers’ *safety*, *performance*, and *wellbeing*. The Research Topic provides a timely analysis of the changing landscape of industrial HRI as we stand on the cusp of a new era in industrial automation, defined by the fusion of human ingenuity and robotic efficiency. The contributions within offer practical insights and forward-thinking perspectives on how collaborative robotics can transform industrial workspaces in the future, in addition to reflecting state-of-the-art research in the field. A different aspect of this intricate relationship is covered by each article in this Research Topic, from the social and psychological effects of incorporating robots into human-centered work environments to the complexities of design and implementation. Developing solutions that are both technologically sophisticated and human-centered requires a holistic approach, which is crucial for comprehending the complex nature of HRI.

Before delving into the particulars of each contribution, we invite the reader to this brief summary, briefly presenting each contribution to the Research Topic through the lenses of safety, performance, and wellbeing. We hope that this will support reflections on the wider societal implications of HRC development, in addition to their technical and ergonomic aspects. A harmonious balance between human needs and machine capabilities will be key to the future of industry.

## 2 Safety

In the field of Human Factors and Cognitive Ergonomics, introducing advanced collaborative robotic systems in production environments necessitates reevaluating safety from different perspectives, namely safety perceptions of workers, safety behaviours and mechanical safety. Integrating this technology in various industrial environments, such as manufacturing and logistics, prompts a critical examination of the interplay of the different elements interacting in the socio-technical system. As with any human-system interaction in the work context, a more ergonomic and anthropocentric system (characteristics that can be measured through optimisation of associated cognitive factors) implies greater safety in terms of prevention and mitigation of potential mechanical risk (understood as collisions, crushing, entrapment, etc.) and psychosocial risk as defined by Occupational Safety and Health Administration (OSHA) such as excessive workload, lack of control, job insecurity or insufficient communication. The present Research Topic includes diverse studies, each exploring different aspects of safety in human-robot collaboration.

The contribution by Mirnig et al. constitutes an excellent opening to the Research Topic. While focusing on automated material handling vehicles, Mirnig et al. discuss many design aspects that are applicable also to HRI more broadly, including contextual factors such as purpose and context of use, and many aspects of the interaction itself. The study by Onnasch et al. investigates how directing a worker’s attention to specific targets with gaze communication can improve safety in human-robot interaction by, first of all, suggesting how robotic eye design could affect operator attention and perceived cognitive workload. Furthermore, the paper indirectly suggests how robotic eyes could potentially prevent mechanical risks like collisions and entrapments. According to research, an operator’s situational awareness and capacity to anticipate and respond to possible hazards are enhanced when they focus on anthropomorphic robot eyes. This study highlights anthropomorphism’s contribution to improving operator safety and attention, leading to safer and more conscious HRIs in industrial settings. On the effect of anthropomorphic features in collaborative robots, the paper by Roesler examines the impact of anthropomorphic versus technical framing of robots on operators’ trust, particularly in the context of robot failures. The study concludes that although the general levels of trust between technically framed and anthropomorphically framed robots did not significantly differ, people perceived the anthropomorphically framed robots as being more transparent, particularly after understandable failures. Because it improves operators’ awareness and skill in anticipating and responding to potential mechanical risks like collisions or entrapments, this increased perceived transparency and positive perception in the event of understandable failures by potentially contributing to increased safety in HRIs. In a complementary way, Freire et al. also addresses the importance of safety in human-robot collaboration, but through a different mechanism. Their proposed cognitive architecture incorporates a “Socially Adaptive Safety Engine,” which dynamically adjusts safety parameters like distance and robot speed based on the worker’s trust level and preferences. While Roesler’s study emphasizes how transparency in robot behavior following failures can enhance safety, Freire et al. go further by actively modifying robot behavior in real-time to adapt to each worker’s trust and comfort, creating a more personalized and context-sensitive safety environment. Together, these articles suggest that fostering both transparency and adaptability in robots—through anthropomorphic design and context-aware systems—can significantly enhance operator safety and wellbeing in industrial environments.

In a comprehensive perspective, Heinold et al. discusses various occupational safety and health (OSH) risks and benefits associated with the integration of robotic systems in industrial settings. These include both physical risks, such as collisions and mechanical failures, and psychosocial risks, including mental stress and job insecurity, which can arise from the use of advanced robotics in workplaces. The study also explores opportunities, such as the potential for reducing physical strain and improving long-term physical health by automating physically demanding tasks. The peculiarity of this manuscript lies in its comprehensive analysis of both physical and psychosocial OSH risks and opportunities, uniquely incorporating workers’ expectations alongside evidence from the literature, offering a dual perspective on the safety implications of HRI. On a similar note, also addressing logistics and agricultural domains in addition to the manufacturing one, Pietrantoni et al. investigated experts’ opinions regarding collaborative robotics safety considerations. Their study emphasized the critical role of tailored safety protocols, highlighting the need for advanced collision avoidance systems, failsafe mechanisms, and emergency stop protocols. Key aspects in agriculture include stability control and navigation on uneven ground for the safety and efficiency of workers. This sectoral approach completes the dual perspective taken by Heinold et al. in that it details how diverse industrial working contexts require tailor-made safety solutions to address both physical risks and ergonomic challenges and further promote the safe integration of robotics into complex work environments.

The impact of human autonomy and robot work pace on job quality in collaborative settings is examined by Van Dijk et al.. They find that higher human autonomy levels correlate with lower perceived workloads. The present article generally addresses some of the main working conditions leading to psychosocial risks according to OSHA, namely excessive workloads, lack of involvement in making decisions that affect the worker, and lack of influence over the way the job is done. This study shows that increasing human autonomy and modifying robot work pace can effectively reduce cognitive and temporal demands on workers. It compares scenarios of human-led work, fast-paced robot-led work, and slow-paced robot-led work. According to these results, reducing workload is linked to a lower mechanical risk because there is a lower probability of mistakes in HRI. This suggests that such measures optimise perceived workload and improve safety in collaborative scenarios.

In the context of an industrial defect inspection task, the article of Cymek et al. examines the phenomenon of decreased individual effort and attention in human–robot collaborative tasks. The study finds that individuals searching for defects with a robot partner may have been less focused and exerted more mental energy than those searching alone, who on average, found more defects. Because less alert workers may be more likely to overlook safety hazards in their environment. This lower level of attentiveness and operational performance in human-robot teams affects productivity and may increase exposure to mechanical risks.

Pluchino et al. examines how collaborative tasks involving robots affect senior workers’ mental workload. The article’s relevance is critical, considering that collaborative robotics is one of the most promising technology for retaining the ageing workforce and maintaining an appropriate quality of work. It finds that senior workers have a strong acceptance of technology and positive experiences during increased cognitive demand. As a result of increased mental demand during dual-task collaboration, the study found that task errors and duration increased despite these favourable perceptions. This might have detrimental effects on safety behaviours. While senior workers are generally open to working with robots, this increased cognitive workload—as indicated by eye tracking and cardiac activity—indicates that overburdening from collaboration may result in overwork and increase the mechanical risks in the workplace.

## 3 Performance

For human-robot interaction to be considered successful, assessing and supporting the performance of the system as a whole is of utmost importance. In fact, one might even say that successful performance of the system is a necessary requisite when arguing for its existence. Successful performance can be defined in many different ways but in essence it is the combination of two things; doing things accurately (effective), and being efficient while doing it. In the context of collaborative human-robot settings, this Research Topic investigates relations between human-factors and performance in terms of temporal performance and cognitive load (Van Dijk et al.; Pluchino et al.), collaborative setting and error rate (Cymek et al.), as well as collaborative setting and perceived workload (Van Dijk et al.). While all these papers are mentioned above in relation to safety, they also bring relevant results in relation to performance.

Van Dijk et al. show a positive correlation between temporal performance and cognitive load, comparing two conditions with a fast vs. slow scheduling for the HRC setup. Pluchino et al. analyze the performance in terms of errors and time on task of senior workers engaged in a sequential collaborative manufacturing task together with a cobot. A dual task condition where the subjects were challenged with a secondary mathematical assignment is compared to a single task (control) condition. Results show that the dual task condition lead to increases in both errors and time spent on task, which corresponded with higher levels of perceived mental effort. However, no differences in perceived performance, as assessed by the NASA-TLX questionnaire, were found between the conditions. Cymek et al. compares two versions of an inspection task, one collaborative where a human operator is working together with a robot, and one individual where the operator is working alone. Results show lower performance for the collaborative setting in terms of fewer identified defects during inspection, indicating an reduction in cognitive load compared to the individual condition.

As previously discussed, the effects on performance of different types of collaborative queues are investigated by Onnasch et al.. An indirect argument is made for faster reallocation of attention as a result of naturalistic attentional queues leading to increased performance. This paper also provides a brief argumentation that some queues used to improve collaboration, e.g., legible motion, may directly impact performance in a negative way, while robot eyes does not.

Finally, in their study of technical expert’s opinions of HRC also mentioned earlier, Pietrantoni et al. found that the introduction of collaborative robots is expected to bring improved efficiency and better worker conditions, e.g. as a result of automation of physically demanding operations. While the participants in the study generally held a positive attitude towards collaborative robots, the increased efficiency was also linked to concerns of job displacement and the need for reskilling.

## 4 Wellbeing

A key concern of cognitive ergonomics is to reduce negative effects of work. This also specifically refers to deployed technologies at the workplace, like advanced robotic systems. However, a truly human-centered approach to workplace and technology design aims at developing a person’s personality and fostering individual and organizational health in its broadest sense. A holistic understanding of health goes beyond the physical safety of humans, but includes mental and social wellbeing of humans. In the ever-evolving landscape of human-robot interaction, the integration of advanced robotics to different workplaces, raises critical questions about how the wellbeing of individuals might be affected. This Research Topic includes different publications, each shedding light on different facets of human-robot-interaction and its implications for the human experience thus potentially leading to wellbeing in the long-term.

As mentioned earlier, Heinold et al. address the question which psycho-social consequences are associated with a close interaction between humans and robots. By combining scientific perspectives through a literature review and insights from workers’ expectations, the study provides a holistic view of the implications of task automation via robotic systems. The findings highlight the psycho-social impacts advanced robotics may have on workers. It becomes clear, that the aspects of task design and function allocation as well as the specific interactions design of systems as well as operation and supervision design are relevant sources potentially affecting the specific user experience and the wellbeing of workers in the long run.

When further considering potential psychological effects, assessing traditional workplace factors can be beneficial. From human factors research it is well understood, that the level of job control or autonomy within a given task is a strong determinant for job quality and wellbeing (Van Der Doef and Maes, 1999). This also applies to industrial tasks (Rosen and Wischniewski, 2019). As working tasks are newly allocated between humans and robots, human autonomy levels can change. The investigation of human autonomy and robotic work pace by Van Dijk et al. discussed earlier is also relevant from a wellbeing perspective. The research underscores the significance of autonomy and work pace in shaping job quality, emphasizing the importance of designing collaborative scenarios that prioritize human autonomy and adjustments to the robot’s work pace to optimize workload and enhance overall wellbeing.

Exploring psycho-social effects more on a team level in this Research Topic is done by Cymek et al.. Their contribution focuses on the well-studied phenomenon of social loafing (Cymek et al.). Using a visual-search task, the presented study investigates whether reduced individual effort, the phenomenon in question, which is commonly observed in human teams, also occurs in human-robot teams. The findings suggest that working with a robot team partner may lead to less attentive task execution, highlighting the need to address mental effort and attention allocation in human-robot collaboration to ensure optimal performance and, consequently, wellbeing.

A human-centred technology design can contribute to a positive human-robot interaction and thus ensure a seamless workflow. One very relevant aspect of robot design which is touched upon in research is the application of anthropomorphic design features (Roesler et al., 2021). Two papers of this Research Topic explore the unique effects of anthropomorphic features in human-robot-interaction on different aspect of the distinct interaction quality and user experience. As mentioned earlier, Onnasch et al. examine how the design of predictive robot eyes influences human attention. The results indicate that anthropomorphic features contribute to a smooth interaction experience. Anthropomorphic robotic eyes trigger reflexive attention reallocation, hinting at a social and automatic processing of artificial stimuli, emphasizing the emotional and cognitive impact of such interactions on wellbeing. Through their analysis of anthropomorphic framing discussed earlier, Roesler show that an adequate level of trust within human-robot-interaction is also an important element contributing to a smooth interaction and a human-centered design. In this paper the perceived transparency of anthropomorphic robots emerges as a key factor, underscoring its role in shaping individuals’ wellbeing.

A novel design approach in order to facilitate socially adaptive robot behaviour in industrial settings is presented by Freire et al.. The authors present a theoretical cognitive architecture for robotic actions control, highlighting modules that among others take into account human preferences and situational awareness and by thus can adapt to human needs. The presented cognitive architecture is integrated into a recycling plant use case for disassembly tasks showcasing the basic functionalities of the systems. In the piloted use cases, the architecture demonstrated key functionalities, such as turn-taking, personalized error-handling, adaptive safety measures, and gesture-based communication, making collaboration smoother and more efficient. The idea of incorporating human preferences and adapting to human needs already on a robot control level can be a promising way to enhance the overall human wellbeing in human-robot interaction.

## 5 Conclusion

In conclusion, this topic underscores the importance of Human Factors and Cognitive Ergonomics in the design and implementation of advanced industrial HRIs. The integration of robotics into industrial settings presents both opportunities and challenges, particularly in enhancing safety, performance, and worker wellbeing. Collaborative robotics can improve productivity and alleviate physical strain on workers, but it also raises concerns about psychosocial risks and job displacement.

The studies included in this Research Topic explore various dimensions of HRI, from safety concerns such as mechanical and psychological risks, to the cognitive demands placed on workers in collaborative environments. It highlights the need for designs that balance technological advancements with human-centric approaches, ensuring safety and wellbeing are not compromised in the pursuit of efficiency.

These papers collectively highlight the elaborate dynamics of human-robot interaction and its different facets each potentially contributing to an overall positive and smooth interaction quality which then eventually is related to an individual’s wellbeing. These studies emphasize the importance of considering human factors at different stages, not only the design phase but also the implementation stage as well as considering newly designed working tasks carefully to ensure a positive impact on individual wellbeing in the workplace.

Future developments in HRI should prioritize interdisciplinary collaboration to develop solutions that consider both human and machine capabilities, promoting an adaptable, efficient, and safer industrial workspace. As industries evolve, a comprehensive understanding of the interaction between humans and robots will be essential for sustainable and productive future workplaces.
